# Evaluating the Impact of Insurer-Sponsored Health Coaching on Hospitalization and Costs in India: A Retrospective Cohort Study

**DOI:** 10.7759/cureus.102823

**Published:** 2026-02-02

**Authors:** Gouri Karajgi, Rajesh Patidar, Khushboo Aggarwal, Kapil Verma

**Affiliations:** 1 Health, Aditya Birla Health Insurance, Gurugram, IND; 2 CEO and Founder, Zyla Health, Gurgaon, IND; 3 Psychology, Private Practice, Gurugram, IND

**Keywords:** claim reduction, health coaching, health insurance, high risk patient care, hospitalization prevention, patient engagement, preventive treatment, wellness

## Abstract

Health coaching programs are emerging as a promising intervention to improve disease management and reduce healthcare costs, particularly in the context of insurer-sponsored wellness initiatives. While such programs have shown positive outcomes in Western healthcare settings, their effectiveness in India remains underexplored. This study examines the impact of a health coaching program implemented by a prominent Indian health insurer (Aditya Birla Health Insurance), focusing on hospitalization rates and claims paid among high-risk policyholders. To assess the intervention's effectiveness, a retrospective analysis was conducted on participants enrolled in the ABHI-Zyla Health Coaching Program from April 2023 to March 2024. Analyzing patient engagement data for 29,463 patients, we found that higher participation in health coaching, measured by the number of interventions made, was associated with lower total claims paid for preventable conditions. Regression results indicate that this relationship is stronger for females and those with a higher number of resolved symptoms as a result of the interventions. These findings suggest that structured health coaching can play a vital role in reducing hospitalization rates and healthcare costs. By providing empirical evidence on the benefits of insurer-led coaching programs, this study offers actionable insights for insurers, policymakers, and healthcare providers. Strengthening such initiatives can contribute to a shift toward proactive disease management, ultimately addressing the growing burden of non-communicable diseases in India, as well as saving claim costs for insurers.

## Introduction

India is witnessing a rapid rise in non-communicable diseases (NCDs), which now account for more than 60% of total deaths. Conditions such as cardiovascular disease, diabetes, and hypertension are placing an immense burden on the healthcare system. This growing prevalence of chronic illnesses is closely linked to rapid urbanization, sedentary lifestyles, poor dietary habits, and an aging population. Data show that the proportion of cardiovascular disease-related deaths nearly doubled from 15% in 1990 to 28% in 2016, marking an 87% relative increase [1]. Much of this escalation can be attributed to preventable complications arising from inadequate disease management, delayed interventions, and lifestyle-related risk factors. India’s constrained public health ecosystem and limited follow-up care further worsen the situation, contributing to higher hospitalization rates - the proportion of users who experienced at least one hospitalization during a specified observation period - and increased healthcare expenditure [2].

Because a significant portion of healthcare expenses in India are paid out-of-pocket, many households experience financial strain or fall into poverty when dealing with chronic illnesses [3]. This financial vulnerability has prompted insurers such as Aditya Birla Health Insurance (ABHI) to explore proactive healthcare strategies aimed at improving patient outcomes while containing costs. Instead of relying solely on reactive medical care, insurers are increasingly turning toward preventive interventions as a means to reduce claims and enhance policyholder well-being.

Despite the growing burden of chronic disease in India, health insurance frameworks have historically adopted a reactive, curative stance, primarily covering hospitalization and treatment costs rather than prevention or disease management. While global evidence demonstrates that health coaching interventions improve clinical outcomes in diabetes, hypertension, and related conditions (reducing HbA1c, blood pressure, and cholesterol levels) while lowering healthcare utilization and costs, such proactive, integrated disease management models remain underexplored within India's insurance landscape. Recent systematic reviews highlight that health coaching significantly enhances quality of life and self-efficacy in chronic illness care, and telemedicine-enabled coaching strategies have proven equally or more effective than in-person consultations in reducing HbA1c by 0.31-0.44% compared to standard care alone. However, most published evidence emanates from developed health systems with different socioeconomic, cultural, and operational contexts. The Indian health insurance sector, despite schemes like Ayushman Bharat and earlier programs such as Rashtriya Swasthya Bima Yojana, has focused primarily on financial protection and hospitalization coverage, with limited empirical evidence on the effectiveness of structured health coaching programs tailored to high-risk, insured populations. This gap is particularly significant given India's escalating diabetes and hypertension prevalence and the cost-containment imperatives facing private insurers.

In recent years, progressive health insurers like ABHI have introduced wellness programs designed to encourage healthy behavior and improve overall health outcomes. However, most initiatives have traditionally centered around physical activity tracking, health screenings, and reward-based incentives [4]. Health coaching - although not uniformly defined in literature - has emerged as a patient-centered approach grounded in established behavior change models such as the Transtheoretical Model, Social Cognitive Theory, and Motivational Interviewing [5]. Typically, health coaching incorporates structured goal setting, personalized guidance, self-monitoring, and accountability, all aimed at sustaining long-term improvements in health behaviors [6].

While the benefits of insurer-sponsored health coaching programs have been documented in Western countries, including reductions in weight, improvements in glycemic control, and smoking cessation [7], their effectiveness in the Indian context remains underexplored. Early evidence from lifestyle interventions in India suggests promising potential for improving chronic disease management [8], yet systematic evaluations of insurer-led health coaching and its influence on hospitalization rates and claims remain limited.

ABHI is among the first insurers in India to launch a structured risk and disease management program targeting high-risk policyholders, initiated around 2020 as part of the broader “Health-First” model and scaled substantially by FY25. Engagement in this program refers to a policyholder’s active participation in health coaching, defined as successful completion of onboarding and, where applicable, continued interaction through scheduled care calls and chat communication, while onboarding denotes the user’s status with respect to informed consent and initiation of the health coaching program, operationalized as successful completion of the initial onboarding call. Through the Chronic Care Management Program and a broader wellness ecosystem, including the WellBeing Score™, HealthReturns™, Healthy Heart Score™, and Active Dayz™, policyholders with chronic conditions such as diabetes, hypertension, hyperlipidemia, and asthma received personalized health coaching from day 1, along with coverage for outpatient diagnostics, consultations, and pharmacy expenses. By FY25, more than one lakh customers had received health coaching services, and another 1.6 lakh had benefited from additional clinical interventions, with 81% of engaged customers showing measurable improvements in key health indicators such as HbA1c, cholesterol, blood pressure, and blood sugar levels. These outcomes illustrate ABHI’s transition from a reactive insurance model to one that proactively manages disease burden and incentivizes long-term healthy behavior, further reinforced by ABHI’s partnership with Zyla Health in 2022 to scale health coaching for specific cohorts of high-risk members.

The present research, conducted using real-world evidence provided by ABHI in collaboration with Zyla Health, seeks to generate actionable insights into how insurers can optimize the design and delivery of health coaching programs. The findings have the potential to inform strategic decisions regarding investments in preventive health initiatives and support a broader shift toward proactive disease management in India’s insurance landscape. Moreover, as India continues to strengthen its public health infrastructure, the insights from this study may guide policymakers in conceptualizing scalable interventions to curb the rising burden of NCDs [9].

The primary objective of this study is to evaluate the impact of an insurer-sponsored health coaching program on total claims paid among high-risk policyholders. Specifically, the analysis will examine how demographic factors such as age and gender influence program adherence and claim outcomes. We expect that higher levels of engagement with coaching sessions will be associated with improved health outcomes and lower hospitalization claims. Additionally, we anticipate that improvements in symptoms may mediate the relationship between coaching engagement and healthcare costs. By exploring these questions, this study aims to contribute evidence-based guidance for insurers, healthcare providers, and policymakers seeking to design more effective preventive health strategies within India’s evolving healthcare ecosystem.

Research questions

This study examines the effect of insurer-led health coaching on healthcare use and costs among high-risk policyholders and addresses the following questions:

What is the impact of insurer-sponsored health coaching on hospitalization rates and claims paid among high-risk policyholders in India?

How do demographic factors (age, gender) influence program adherence and hospitalization outcomes?

Is higher engagement with coaching sessions (measured by number of calls) associated with improved health outcomes and lower hospitalization rates?

Do symptom improvements mediate the relationship between coaching engagement and healthcare costs?

How does gender moderate the relationship between coaching call frequency and total claims paid?

How does symptom resolution (both complete and partial) moderate the relationship between coaching calls and healthcare expenditure?

## Materials and methods

ABHI offers a comprehensive health coaching program designed to support high-risk policyholders who are living with chronic conditions such as dyslipidemia, hypertension, type 2 diabetes, and obesity. The program seeks to promote sustained health behavior change and reduce the likelihood of complications that frequently lead to hospitalization. To ensure broad awareness, ABHI promotes the program through its agent network as well as its digital platforms, allowing beneficiaries across demographic groups to learn about and access the service. The initiative is jointly developed and overseen by ABHI and Zyla Health, enabling the integration of clinical expertise with technology-driven engagement to deliver personalized and structured interventions. High-risk individuals are identified using underwriting data, historical claims information, and findings from annual health check-ups. Once flagged, trained representatives reach out by phone to explain the program and onboard eligible members.

After enrollment, patients enter a structured nine-month coaching journey consisting of six scheduled sessions with a qualified clinical nutritionist. Reminder notifications are sent one day before and on the day of each appointment to promote adherence. The first coaching session, which is conducted in the patient’s regional language, acts as a detailed intake assessment. During this call, the health coach captures information on the patient’s medications, presenting symptoms, coexisting conditions, laboratory results, previous surgeries, family health history, lifestyle habits, and daily routines. All details are systematically documented in ABHI’s in-house electronic medical record (EMR) system to ensure consistency and continuity in follow-up interactions. Drawing from this assessment, the coach sets individualized health goals with the patient and formulates a customized nutrition plan that reflects the patient’s medical needs, demographic profile, and food preferences. Follow-up coaching sessions take place approximately every six weeks and are focused on reviewing adherence, monitoring changes in symptoms, and reinforcing motivation to maintain lifestyle improvements. Medical questions raised during these sessions are addressed by a general physician, while issues such as body pain are escalated to a physiotherapist for further guidance.

Alongside these scheduled sessions, the program incorporates personalized digital engagement delivered through ABHI’s omni-channel platforms. Patients receive targeted nudges and reminders to track specific health parameters, along with curated educational content tailored to their primary chronic condition. Additional support is available through ABHI’s integrated mobile application, where patients can submit queries at any time, ensuring continuous guidance beyond formal coaching sessions. This combination of structured coaching, medical oversight, and technology-based reinforcement supports long-term engagement and strengthens disease management outcomes.

To evaluate the effectiveness of this intervention, we conducted a retrospective analysis of individuals enrolled in the ABHI-Zyla Health Coaching Program between April 2023 and March 2024. The study draws from two primary data sources. The first includes information supplied by ABHI that is available at the time of underwriting, along with claims data, results from annual health assessments, and any newly declared health information submitted during the policy period. This information is securely transferred to the coaching team through system integrations and data-sharing mechanisms that comply with applicable security regulations. The second source consists of health-related data collected during the coaching journey itself, gathered through regular coach interactions and digital touchpoints within ABHI’s app. All data are coded using standardized medical classifications such as ICD-10 and LOINC to ensure accuracy, consistency, and analytical reliability.

Participants were eligible for inclusion in the study if they were registered members of ABHI who had been identified as high-risk for health complications associated with chronic conditions. Only individuals who had at least one diagnosis of obesity, type 2 diabetes, dyslipidemia, or hypertension were considered, and they must have been enrolled in the ABHI-Zyla Health Coaching Program between April 2023 and March 2024. Participants were excluded if they had incomplete or missing baseline data required for analysis, were diagnosed primarily with acute or non-chronic conditions beyond the scope of the coaching program, withdrew consent for the use of their data, or had records that could not be reliably linked across the ABHI and Zyla data systems. The final analytical sample comprised 29,463 patients who had at least one diagnosis of obesity, type 2 diabetes, dyslipidemia, or hypertension.

Symptoms were recorded using the International Classification of Diseases, Tenth Revision (ICD-10) framework (Appendix). At enrollment, symptoms were identified through a structured telephonic interaction conducted by a trained health coach and coded into ICD-10 based on patient-reported complaints and clinician-approved mapping protocols embedded within Zyla Health’s electronic medical record system. During each follow-up interaction, the health coach systematically assessed the status of previously recorded symptoms using a standardized questionnaire. Symptom status was determined based on patient self-report during the phone consultation and categorized into one of four predefined outcome states: same as before, better than before, completely resolved, or worse than before. The categorization criteria were as follows: “completely resolved” indicated that the patient reported complete absence of the symptom since the prior interaction; “better than before” indicated a clear subjective improvement in symptom frequency, intensity, or functional impact, but with persistence of the symptom to some degree; “same as before” indicated no meaningful change in the symptom as perceived by the patient; and “worse than before” indicated an increase in symptom severity, frequency, or negative impact on daily activities compared to the prior assessment. Symptom resolution and improvement status were therefore based on patient self-report rather than direct clinical examination. Health coaches were trained to use consistent probing questions and documentation standards to minimize inter-observer variability, and symptom status was recorded in the electronic medical record.

Our measurement strategy focused on capturing the key elements of program engagement and health progression. Onboarded status was recorded as a binary indicator reflecting whether a patient consented to participate in the coaching program and actively began using the service. Engagement was further assessed through the number of successful calls completed with the health coach following onboarding. Health status at baseline was captured through the total number of symptoms reported during the initial assessment call. Measures of improvement included the number of symptoms that had fully resolved and the number that had improved but not completely resolved, both documented in the EMR system. Demographic attributes such as age and gender were included to account for their potential influence on engagement and outcomes.

The study’s dependent variables focused on healthcare utilization following the intervention. The total number of preventable claims post-intervention captured claims linked to complications from chronic conditions that could potentially be avoided with better disease management, excluding unavoidable medical events such as those involving trauma or maternity care. The total claim paid amount for such preventable claims captured the financial impact on the insurer. When examined together, these variables offered a comprehensive understanding of how program participation and symptom changes relate to healthcare use and cost.

The analysis aimed to address three main objectives. First, we examined correlations among the independent variables, demographic characteristics, and the two key dependent outcomes. Second, we evaluated the direct effects of age, gender, onboarding status, engagement (measured through number of calls), and symptom-related indicators on the total claim paid amount for preventable conditions after the intervention. Finally, given that the number of coaching calls represents the core mechanism through which patients receive support, we tested whether its influence on claim costs was moderated by key variables such as gender and symptom improvements, including both complete resolution and partial improvement. Together, these analyses provide deeper insights into the pathways through which health coaching may influence patient outcomes and healthcare expenditures.

## Results

Prior to analysis, data completeness was assessed across all variables. Of the 38,250 eligible participants, 8,787 (22.9%) were excluded due to missing outcome data (total claims post-intervention) or incomplete baseline demographic information. The final analytical sample comprised 29,463 individuals (77.1%). Missing data patterns were examined; data were found to be missing completely at random (MCAR) as assessed using Little's MCAR test (χ² = 124.5, p = 0.053). Given the large sample size and assumption of MCAR, complete-case analysis was deemed appropriate without employing multiple imputation.

Descriptive statistics (means, standard deviations, and frequency distributions) for all variables were computed. Pearson correlations were calculated to assess bivariate associations among continuous variables and point-biserial correlations for categorical-continuous relationships. Normality of the dependent variable (total claim paid amount) was assessed using Q-Q plots and the Shapiro-Wilk test. Given significant departures from normality (W = 0.823, p < 0.001), we log-transformed the outcome variable to satisfy regression assumptions, while unstandardized estimates were back-transformed for interpretation.

The following three hierarchical ordinary least squares (OLS) regression models were fitted with total claim paid amount for preventable claims (post-intervention) as the dependent variable:

Model 1 (baseline): age, gender, and onboarded status as predictors

Model 2: added engagement (number of calls) and symptom-related variables

Model 3: tested moderation effects by including interaction terms (number of calls × gender; number of calls × symptoms resolved; number of calls × symptoms better)

Analyses were conducted in R (version 4.3.1) using the car, lmtest, and interactions packages. All statistical tests were two-tailed with α = 0.05.

Table 1 presents the mean values, standard deviations, and correlations (unadjusted) among the study variables for 29,463 patients (77.1%). The average age of patients in the sample was 42.1 years, and 10,607 (36%) of the participants were female. Age demonstrated a positive and significant correlation with all other variables in the study, indicating that older patients tended to engage more with the program and reported a greater number of symptoms, as well as more symptoms that had improved or resolved. Consistent with this pattern, we found that the average age of onboarded patients (43.4 years; N = 15,077) was significantly higher than that of patients who did not onboard (40.8 years; N = 14,386), a difference that was statistically significant (p < 0.001).

**Table 1:** Means, SDs, and correlations among the study variable Gender – 0 as male and 1 as female. Onboarding status – 0 as not onboarded and 1 as onboarded. #Total claim paid amount for preventable claims post-intervention (N = 29,463). *p < 0.05. SD, standard deviation

S. No.	Variables	Mean	SD	1	2	3	4	5	6	7	8
1.	Age	42.1	10.3	-	-	-	-	-	-	-	-
2.	Gender	0.36	0.48	0.07*	-	-	-	-	-	-	-
3.	Onboarded status	0.51	0.50	0.12*	-0.07*	-	-	-	-	-	-
4.	No. of calls	1.12	1.45	0.10*	-0.07*	0.75*	-	-	-	-	-
5.	No. of symptoms	0.69	1.38	0.07*	0.07*	0.48*	0.50*	-	-	-	-
6.	No. of symptoms better	0.12	0.47	0.04*	0.04*	0.24*	0.35*	0.52*	-	-	-
7.	No. of symptoms resolved	0.14	0.53	0.02*	0.01	0.26*	0.44*	0.55*	0.21*	-	-
8.	No. of preventable claims	0.01	0.09	0.04*	0.00	0.07*	0.00	0.00	0.00	0.00	-
9.	Total claim^#^	701.1	13969.5	0.04*	-0.01*	0.05*	0.01	-0.01	0.00	-0.01	0.64*

Gender was also significantly associated with several program engagement and health outcome indicators. Female participants reported a higher number of symptoms compared to male participants but made fewer calls to the health coach. The proportion of females in the not-onboarded group (N=11,638; 39.5%) was significantly higher than in the onboarded group (N=9,664; 32.8%), suggesting lower levels of uptake among women (p < 0.001). Onboarding status itself was found to be higher among male participants and was positively correlated with the total claims paid amount. This association is likely driven by selection bias, as individuals with more severe health conditions - who naturally incur higher claim amounts - are more likely to opt into the coaching program. Importantly, the number of coaching calls made by patients was positively correlated with the number of symptoms reported initially, as well as the number of symptoms that improved or resolved during the course of the program, indicating that higher engagement is associated with better health progress.

In terms of overall program engagement, 15,085 (51.2%) of patients with at least one chronic diagnosis onboarded into the program by providing consent during the outreach call. Among those who onboarded, only 4,963 (32.9%) were female, echoing earlier gender-based differences in participation. Nearly half of the full sample (7,497; 49.7%) made at least one call with the health coach. For onboarded members, the average number of calls was 2.2, with a median of two calls, suggesting moderate engagement levels across the cohort. The mean number of claims post-enrollment was 1.02 in engaged users versus 1.48 in non-engaged users (p<0.05), representing a 31% reduction in healthcare utilization events among those actively participating in the coaching program.

Table 2 summarizes the regression analyses (adjusted) conducted to identify factors influencing the total claim paid amount for preventable claims following the intervention. We also tested moderation effects of gender, number of symptoms resolved, and number of symptoms improved on the relationship between the number of calls and total claims paid. Age emerged as a significant predictor of claim amounts, with older patients incurring higher total claims compared to younger individuals (b = 0.03; p < 0.01). After controlling for age and gender, the number of calls showed a significant negative association with total preventable claim amounts (b = -0.08; p < 0.01), indicating that increased engagement through coaching calls is linked to reduced healthcare costs.

**Table 2:** Results of regression analysis Note: N = 29,463 SE, standard error; b, standardized estimate; no. of calls, number of calls; no. of symptoms, number of symptoms; no. of symptoms better, number of symptoms better; no. of symptoms resolved, number of symptoms resolved *p < 0.05; **p < 0.01.

	Total claim paid amount for preventable claims post-intervention					
	Model 1		Model 2		Model 3	
Predictors	(1)b	SE	b	SE	b	SE
Intercept	-0.11**	0.01	-0.10**	0.01	-0.11**	0.01
Age	0.03**	0.01	0.03**	0.01	0.03**	0.01
Gender	-0.01	0.01	-0.01	0.01	-0.01	0.01
Onboarded status	0.21**	0.00	0.21**	0.02	0.21**	0.02
No. of calls	-0.08**	0.01	-0.08**	0.00	-0.08**	0.01
No. of symptoms	-	0.00	-	0.01	-	0.01
No. of symptoms better	-	-	-	-	-0.02	0.01
No. of symptoms resolved	-	-	-0.01	0.01	-	-
No. of calls * gender	-0.02*	0.01	-	-	-	-
No. of calls * no. of symptoms resolved	-	-	0.01*	0.00	-	-
No. of calls * no. of symptoms better	-	-	-	-	0.02*	0.01

Further insights from the unstandardized regression coefficients in Table 3 reveal that each additional coaching call is associated with a reduction of INR 667.99 in total claim amounts, holding all other variables constant. When accounting for the average cost of delivering the intervention, we found that every rupee invested in coaching calls yielded savings of approximately 1.72 rupees in claim payments. The analysis was conducted on a sample of 29,463 individuals with at least one of the four chronic conditions under focus, among whom 155 preventable hospitalization claims were recorded in the post-intervention period.

**Table 3:** Results of regression analysis (unstandardized) Note: N = 29,463 SE, standard error; b, unstandardized estimate *p < 0.01.

	Total claim paid amount for preventable claims post -intervention	
Predictors	b	SE
Intercept	-1609.25*	344.33
Age	40.82*	7.89
Gender	-134.95	171.26
Onboarded status	3052.78*	251.54
No. of calls	-667.99*	87.82
No. of symptoms	-257.457*	69.96

**Figure 1:**
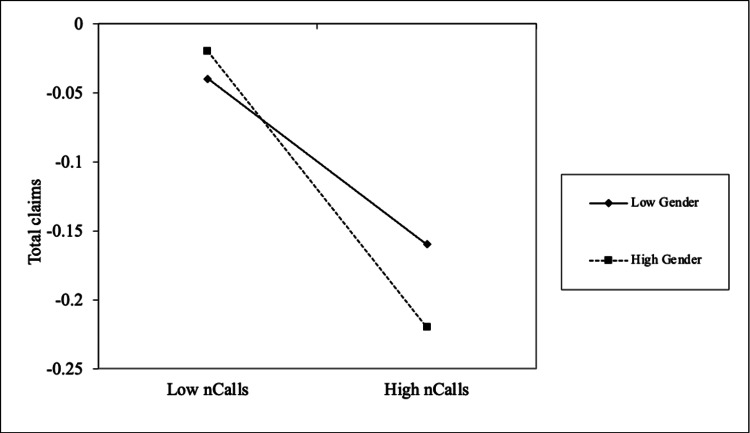
The interaction effects of gender on the relationship between no. of calls and total claims paid amount *Total claims: total value (in Indian rupees) of preventable claims paid post-intervention *nCalls: number of successful coach calls to a user *Low gender: males; high gender: females

Gender significantly moderated the relationship between the number of calls and the total claim paid amount (b = -0.02; p < 0.05). Specifically, the negative association between coaching calls and claim amounts was stronger for female patients. Conditional effects computed at high (+1 SD) and low (-1 SD) levels of gender, along with simple slope tests [10], revealed that increases in the number of calls resulted in a significantly greater reduction in claim amounts for women compared to men, as illustrated in Figure 1.

**Figure 2:**
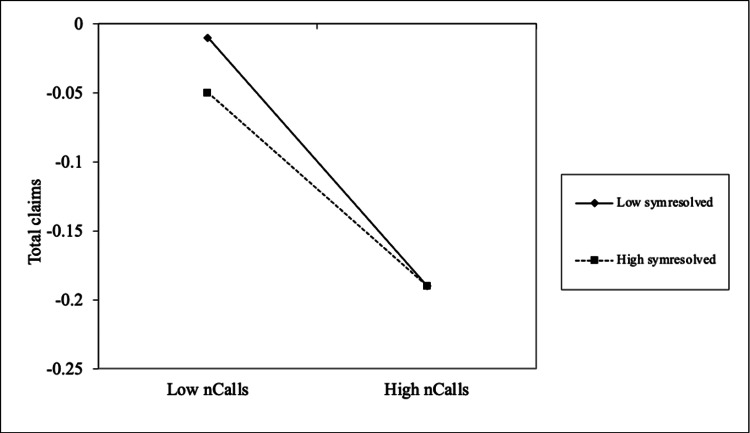
The interaction effects of no. of symptoms resolved on the relationship between no. of calls and total claims paid amount *Total claims: total value (in Indian rupees) of preventable claims paid post-intervention *nCalls: number of successful coach calls to a user *symresolved: symptoms resolved after intervention

The number of symptoms resolved also moderated the relationship between call frequency and claim amounts (b = 0.01; p < 0.05). Patients who experienced a higher number of resolved symptoms benefited more from increased engagement, showing a stronger negative relationship between calls and claim amounts. As shown in Figure 2, individuals with a greater number of resolved symptoms incurred lower claim amounts across both low and high levels of call frequency. Among the 4,272 unique users who showed symptom improvement or resolution, a total of 7,626 symptoms were documented as improved or resolved.

**Figure 3:**
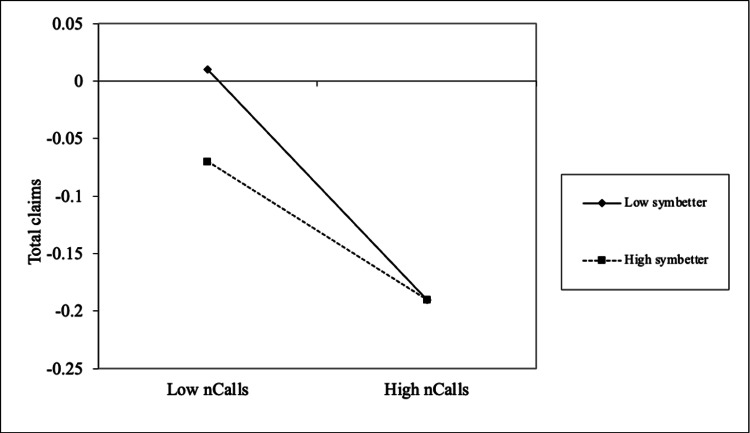
The interaction effects of no. of symptoms better on the relationship between no. of calls and total claims paid amount *Total claims: total value (in Indian rupees) of preventable claims paid post-intervention *nCalls: number of successful coach calls to a user *symbetter: symptoms better after intervention

Finally, the number of symptoms reported as "better" (but not fully resolved) significantly moderated the relationship between the number of calls and total claims (b = 0.02; p < 0.05). As illustrated in Figure 3, patients with fewer partially improved symptoms demonstrated a steeper reduction in claim amounts as call frequency increased. Nonetheless, individuals with a higher number of improved symptoms consistently reported lower claim amounts regardless of whether they made relatively few or many calls.

## Discussion

The findings of this study provide valuable insights into the association between ABHI-sponsored health coaching programs and healthcare costs among high-risk policyholders in India. The results highlight the observed relationship between patient engagement in such programs and health outcomes, with a notable correlation between the number of coaching calls and reported improvements in health status. These findings are consistent with existing literature suggesting a role for structured health coaching in supporting patient self-management and reducing healthcare utilization [5].

One notable finding is that older patients demonstrated higher program engagement, with greater frequency of coaching calls and higher reported rates of symptom resolution. This pattern aligns with research indicating that older adults tend to engage more actively in health interventions, potentially due to increased health concerns and disease management needs [11]. Additionally, age showed a positive association with total claims paid, consistent with the well-established observation that older individuals typically incur higher healthcare costs [12]. These patterns suggest that while health coaching may benefit individuals across age groups, tailored strategies could potentially enhance engagement among younger policyholders, who may perceive a lower immediate need for such interventions. Age was found to be positively correlated with number of calls, number of symptoms reported as better, and number of symptoms reported as resolved. Older patients made more coaching calls and reported higher rates of symptom resolution, while also demonstrating higher overall healthcare costs. These associations may reflect heightened health awareness and greater chronic disease burdens commonly observed in older populations.

Gender differences in engagement patterns and reported outcomes also emerged as a notable theme. While female participants reported a higher number of symptoms, they made fewer calls than male counterparts. This pattern may reflect potential barriers to participation, such as competing household responsibilities or differential health-seeking behaviors. Notably, female participants showed a stronger negative association between the number of calls and total claims paid, suggesting that increased engagement may correspond to greater cost reductions for women. This finding underscores the potential value of gender-informed health coaching strategies that address identified barriers and support participation among women. In the Indian social context, these patterns may reflect broader structural factors, including differential household responsibilities and variable access to medical attention between genders.

The study also reveals an association between symptom resolution and claims reduction. The number of calls made by health coaches showed a positive correlation with both symptom resolution and symptom improvement. Furthermore, symptom resolution appeared to strengthen the association between coaching calls and total claims paid, suggesting that patients reporting symptom relief also demonstrated lower healthcare costs. This pattern is consistent with prior findings documenting associations between improved symptom management and reduced hospitalization rates and overall medical expenditures [13]. These results suggest that greater patient engagement in health coaching programs may correspond with economic benefits, building a case for further exploration of such initiatives by insurers.

An important observation concerns onboarding status, which was positively correlated with total claims paid, likely reflecting selection bias wherein individuals with more severe health conditions are preferentially enrolled in coaching programs and consequently file more claims. This finding is consistent with prior research indicating that high-risk patients are more likely to participate in health interventions due to perceived need for medical support [14]. However, the data also suggest that sustained engagement through multiple coaching sessions corresponds with cost reductions, indicating potential long-term financial and health associations with continued participation.

A particularly notable finding is the negative association between the number of coaching calls and total claims paid for preventable conditions. This association appeared stronger for patients who reported resolution of a greater number of symptoms. This pattern suggests that structured, personalized health coaching interventions may be associated with reduced financial burden related to preventable complications from chronic conditions such as diabetes, hypertension, and cardiovascular diseases. Given the substantial out-of-pocket healthcare expenditure in India, these findings are potentially relevant for both insurers and policymakers. They suggest that expanded access to such preventive programs warrants further investigation for potential public health and economic benefits [15, 16].

The findings of this study suggest a potential shift in India's insurance-led health delivery paradigm: structured health coaching programs may be associated with measurable improvements in health status among high-risk, insured populations, bridging traditional distinctions between insurance coverage and health outcomes. The observed finding that 81% of engaged customers reported improvements in HbA1c, cholesterol, and blood pressure is consistent with patterns documented in global meta-analyses and extends this literature to India's market-driven, private insurance context - an area where evidence remains limited. The mechanisms underlying these observed associations likely involve behavioral pathways described in health coaching theory: personalized goal-setting, continuous monitoring, motivational support, and accountability may support behavioral change and treatment adherence, which, in turn, could be associated with improvements in glycemic and cardiometabolic control. Notably, the engagement definition employed in this study (successful onboarding plus continued interaction) captures an often-unmeasured dimension in effectiveness research: real-world engagement patterns that reflect actual intervention uptake. By operationalizing engagement and examining its association with reported clinical improvement, this study contributes evidence suggesting that coaching-based disease management approaches may be both clinically relevant and operationally feasible at scale in the Indian context. Future research should examine cost-effectiveness outcomes and long-term sustainability trajectories, particularly as India considers scaling such models across its expanding insurance base to address chronic disease burden disparities across income and geographic distribution.

Limitations and future research directions

Despite these promising results, this study has certain limitations. First, the reliance on self-reported symptoms may introduce bias, as patients' perceptions of improvement might not always align with objective clinical assessments. Future research should incorporate biometric and medical test data to validate self-reported outcomes. Second, while the study controlled for key demographic variables such as age and gender, additional factors such as socioeconomic status, educational background, and occupation could influence program engagement and health outcomes. Future studies should explore these aspects to provide a more comprehensive understanding of health coaching effectiveness. Third, the study primarily focused on short-term claims data, and a longitudinal analysis would be necessary to assess the sustained impact of health coaching on long-term healthcare costs and chronic disease progression.

Looking ahead, future research and policy implications should consider integrating health coaching with broader public health initiatives. Given the rapid expansion of India's digital health ecosystem, leveraging mobile health (mHealth) technologies and AI-driven personalized coaching could enhance program scalability and effectiveness. Additionally, insurers could explore incentive structures, such as reduced premiums for active participants, to further encourage policyholder engagement.

## Conclusions

This study provides strong evidence supporting the effectiveness of insurer-led health coaching programs in India. Higher engagement in coaching sessions is associated with improved symptom resolution and reduced healthcare costs, particularly for females and older individuals. These findings highlight the need for targeted strategies to enhance engagement, reduce barriers to participation, and optimize program delivery. By shifting from a reactive to a preventive healthcare model, insurers and policymakers can play a crucial role in mitigating the growing burden of NCDs in India.

## Appendices

**Table 4:** ICD level 1 and tags

ICD level 1	Tags
A00-A09 - Intestinal infectious diseases	Non-preventable
A00-B99 - Certain infectious and parasitic diseases	Non-preventable
A01 - Typhoid and paratyphoid fevers	Non-preventable
A010 - Typhoid fever	Non-preventable
A02 - Other salmonella infections	Non-preventable
A0222 - Salmonella pneumonia	Non-preventable
A04 - Other bacterial intestinal infections	Non-preventable
A06 - Amebiasis	Non-preventable
A07 - Other protozoal intestinal diseases	Non-preventable
A08 - Viral and other specified intestinal infections	Non-preventable
A09 - Infectious gastroenteritis and colitis, unspecified	Non-preventable
A15 - Respiratory tuberculosis	Non-preventable
A15-A19 - Tuberculosis	Non-preventable
A18 - Tuberculosis of other organs	Non-preventable
A1801 - Tuberculosis of spine	Non-preventable
A20-A28 - Certain zoonotic bacterial diseases	Non-preventable
A30-A49 - Other bacterial diseases	Non-preventable
A36 - Diphtheria	Non-preventable
A37 - Whooping cough	Non-preventable
A38 - Scarlet fever	Non-preventable
A40 - Streptococcal sepsis	Non-preventable
A41 - Other sepsis	Non-preventable
A49 - Bacterial infection of unspecified site	Non-preventable
A54 - Gonococcal infection	Non-preventable
A66 - Yaws	Non-preventable
A75-A79 - Rickettsioses	Non-preventable
A80-A89 - Viral and prion infections of the central nervous system	Non-preventable
A83 - Mosquito-borne viral encephalitis	Non-preventable
A90 - Dengue fever [classical dengue]	Non-preventable
A90-A99 - Arthropod-borne viral fevers and viral hemorrhagic fevers	Non-preventable
A92 - Other mosquito-borne viral fevers	Non-preventable
A98 - Other viral hemorrhagic fevers, not elsewhere classified	Non-preventable
Abdominal and pelvic pain (R10)	Non-preventable
Abnormal blood-pressure reading, without diagnosis (R03)	Preventable
Abnormal tumor markers (R97)	Non-preventable
Abnormalities of heart beat (R00)	Preventable
Abscess of anal and rectal regions (K61)	Non-preventable
Absent, scanty and rare menstruation (N91)	Non-preventable
Acc hit, strk, kick, twist, bite or scratch by another prsn (W50)	Non-preventable
Acquired hemolytic anemia (D59)	Preventable
Acute appendicitis (K35)	Non-preventable
Acute bronchiolitis (J21)	Non-preventable
Acute bronchitis (J20)	Preventable
Acute hepatitis A (B15)	Non-preventable
Acute kidney failure (N17)	Non-preventable
Acute pancreatitis (K85)	Preventable
Acute pharyngitis (J02)	Non-preventable
Acute pyelonephritis (N10)	Preventable
Acute sinusitis (J01)	Preventable
Acute tonsillitis (J03)	Preventable
Acute upper resp infections of multiple and unsp sites (J06)	Non-preventable
Adverse effects, not elsewhere classified (T78)	Non-preventable
Age-related cataract (H25)	Non-preventable
Allergy status to drug/meds/biol subst (Z88)	Non-preventable
Amebiasis (A06)	Non-preventable
Angina pectoris (I20)	Preventable
Aphagia and dysphagia (R13)	Non-preventable
Arenaviral hemorrhagic fever (A96)	Non-preventable
Asthma (J45)	Preventable
Atrial fibrillation and flutter (I48)	Preventable
B00-B09 - Viral infections characterized by skin and mucous membrane lesions	Non-preventable
B02 - Zoster [herpes zoster]	Non-preventable
B15-B19 - Viral hepatitis	Non-preventable
B17 - Other acute viral hepatitis	Non-preventable
B25 - Cytomegaloviral disease	Non-preventable
B25-B34 - Other viral diseases	Non-preventable
B26 - Mumps	Non-preventable
B34 - Viral infection of unspecified site	Non-preventable
B35-B49 - Mycoses	Non-preventable
B37 - Candidiasis	Non-preventable
B42 - Sporotrichosis	Non-preventable
B43 - Chromomycosis and phaeomycotic abscess	Non-preventable
B50-B64 - Protozoal diseases	Non-preventable
B51 - Plasmodium vivax malaria	Non-preventable
B66 - Other fluke infections	Non-preventable
B965 - Pseudomonas (aeruginosa) (mallei) (pseudomallei) as the cause of diseases classified elsewhere	Non-preventable
Bacterial meningitis, not elsewhere classified (G00)	Non-preventable
Bacterial pneumonia, not elsewhere classified (J15)	Non-preventable
Bacterial sepsis of newborn (P36)	Non-preventable
Benign lipomatous neoplasm (D17)	Non-preventable
Benign neoplasm of brain and oth prt central nervous system (D33)	Non-preventable
Benign neoplasm of male genital organs (D29)	Non-preventable
Benign neuroendocrine tumors (D3A)	Non-preventable
Bronchitis, not specified as acute or chronic (J40)	Non-preventable
Burn and corrosion of head, face, and neck (T20)	Non-preventable
Burn and corrosion of shoulder/upper limb, except wrist and hand (T22)	Non-preventable
Burns classified accord extent body involvement (T31)	Non-preventable
C00 - Malignant neoplasm of lip	Non-preventable
C00-C14 - Malignant neoplasms of lip, oral cavity and pharynx	Non-preventable
C00-D49 - Neoplasms	Non-preventable
C02 - Malignant neoplasm of other and unspecified parts of tongue	Non-preventable
C029 - Malignant neoplasm of tongue, unspecified	Non-preventable
C06 - Malignant neoplasm of other and unspecified parts of mouth	Non-preventable
C08 - Malignant neoplasm of other and unspecified major salivary glands	Non-preventable
C13 - Malignant neoplasm of hypopharynx	Non-preventable
C15 - Malignant neoplasm of esophagus	Non-preventable
C15-C26 - Malignant neoplasms of digestive organs	Non-preventable
C16 - Malignant neoplasm of stomach	Non-preventable
C169 - Malignant neoplasm of stomach, unspecified	Non-preventable
C17 - Malignant neoplasm of small intestine	Non-preventable
C18 - Malignant neoplasm of colon	Non-preventable
C18. 1 – Carcinoma	Non-preventable
C181 - Malignant neoplasm of appendix	Non-preventable
C24 - Malignant neoplasm of other and unspecified parts of biliary tract	Non-preventable
C25 - Malignant neoplasm of pancreas	Non-preventable
C30-C39 - Malignant neoplasms of respiratory and intrathoracic organs	Non-preventable
C34 - Malignant neoplasm of bronchus and lung	Non-preventable
C41 - Malignant neoplasm of bone and articular cartilage of other and unspecified sites	Non-preventable
C43 - Malignant melanoma of skin	Non-preventable
C44 - Other and unspecified malignant neoplasm of skin	Non-preventable
C45-C49 - Malignant neoplasms of mesothelial and soft tissue	Non-preventable
C46 - Kaposi's sarcoma	Non-preventable
C49 - Malignant neoplasm of other connective and soft tissue	Non-preventable
C50 - Malignant neoplasm of breast	Non-preventable
C50011 - Malignant neoplasm of nipple and areola, right female breast	Non-preventable
C51-C58 - Malignant neoplasms of female genital organs	Non-preventable
C53 - Malignant neoplasm of cervix uteri	Non-preventable
C54 - Malignant neoplasm of corpus uteri	Non-preventable
C56 - Malignant neoplasm of ovary	Non-preventable
C569 - Malignant neoplasm of unspecified ovary	Non-preventable
C60 - Malignant neoplasm of penis	Non-preventable
C60-C63 - Malignant neoplasms of male genital organs	Non-preventable
C64 - Malignant neoplasm of kidney, except renal pelvis	Non-preventable
C64-C68 - Malignant neoplasms of urinary tract	Non-preventable
C67 - Malignant neoplasm of bladder	Non-preventable
C69-C72 - Malignant neoplasms of eye, brain and other parts of central nervous system	Non-preventable
C71 - Malignant neoplasm of brain	Non-preventable
C719 - Malignant neoplasm of brain, unspecified	Non-preventable
C73-C75 - Malignant neoplasms of thyroid and other endocrine glands	Non-preventable
C76 - Malignant neoplasm of other and ill-defined sites	Non-preventable
C76-C80 - Malignant neoplasms of ill-defined, other secondary and unspecified sites	Non-preventable
C78 - Secondary malignant neoplasm of respiratory and digestive organs	Non-preventable
C7A - Malignant neuroendocrine tumors	Non-preventable
C7A - Malignant neuroendocrine tumors	Non-preventable
C80 - Malignant neoplasm without specification of site	Non-preventable
C81 - Hodgkin lymphoma	Non-preventable
C81-C96 - Malignant neoplasms of lymphoid, hematopoietic and related tissue	Non-preventable
C84 - Mature T/NK-cell lymphomas	Non-preventable
C90 - Multiple myeloma and malignant plasma cell neoplasms	Non-preventable
C91 - Lymphoid leukemia	Non-preventable
C92 - Myeloid leukemia	Non-preventable
Calculus of kidney and ureter (N20)	Non-preventable
Calculus of lower urinary tract (N21)	Non-preventable
Candidiasis (B37)	Non-preventable
carcinoma breast - carcinoma breast	Non-preventable
Carcinoma in situ of breast (D05)	Non-preventable
Carcinoma in situ of oral cavity, esophagus and stomach (D00)	Non-preventable
Carcinoma in situ of other and unspecified digestive organs (D01)	Non-preventable
Carcinoma in situ of other and unspecified genital organs (D07)	Non-preventable
Cardiomyopathy (I42)	Preventable
Cellulitis and acute lymphangitis (L03)	Non-preventable
Cerebral infarction (I63)	Preventable
Certain current complications following ST elevation (STEMI) and non-ST elevation (NSTEMI) myocardial infarction ≤ 28 day (I23)	Preventable
Cholecystitis (K81)	Non-preventable
Cholelithiasis (K80)	Non-preventable
Cholera (A00)	Non-preventable
Chronic ischemic heart disease (I25)	Preventable
Chronic kidney disease (CKD) (N18)	Non-preventable
Chronic sinusitis (J32)	Preventable
Chronic viral hepatitis (B18)	Non-preventable
Complications and ill-defined descriptions of heart disease (I51)	Preventable
Complications of internal orthopedic prosthetic devices, implants and grafts (T84)	Non-preventable
Conductive and sensorineural hearing loss (H90)	Non-preventable
Contact with dog (W54)	Non-preventable
Convulsions, not elsewhere classified (R56)	Non-preventable
Cutaneous abscess, furuncle and carbuncle (L02)	Non-preventable
Cystitis (N30)	Non-preventable
D00 - Carcinoma in situ of oral cavity, esophagus and stomach	Non-preventable
D00-D09 - In situ neoplasms	Non-preventable
D01 - Carcinoma in situ of other and unspecified digestive organs	Non-preventable
D05 - Carcinoma in situ of breast	Non-preventable
D10 - Benign neoplasm of mouth and pharynx	Non-preventable
D10-D36 - Benign neoplasms, except benign neuroendocrine tumors	Non-preventable
D11 - Benign neoplasm of major salivary glands	Non-preventable
D17 - Benign lipomatous neoplasm	Non-preventable
D22 - Melanocytic nevi	Non-preventable
D23 - Other benign neoplasms of skin	Non-preventable
D24 - Benign neoplasm of breast	Non-preventable
D25 - Leiomyoma of uterus	Non-preventable
D259 - Leiomyoma of uterus, unspecified	Non-preventable
D27 - Benign neoplasm of ovary	Non-preventable
D29 - Benign neoplasm of male genital organs	Non-preventable
D31 - Benign neoplasm of eye and adnexa	Non-preventable
D32 - Benign neoplasm of meninges	Non-preventable
D35 - Benign neoplasm of other and unspecified endocrine glands	Non-preventable
D36 - Benign neoplasm of other and unspecified sites	Non-preventable
D37-D48 - Neoplasms of uncertain behavior, polycythemia vera and myelodysplastic syndromes	Non-preventable
D43 - Neoplasm of uncertain behavior of brain and central nervous system	Non-preventable
D46 - Myelodysplastic syndromes	Non-preventable
D469 - Myelodysplastic syndrome, unspecified	Non-preventable
D47 - Other neoplasms of uncertain behavior of lymphoid, hematopoietic and related tissue	Non-preventable
D48 - Neoplasm of uncertain behavior of other and unspecified sites	Non-preventable
D50 - Iron deficiency anemia	Preventable
D50-D53 - Nutritional anemias	Preventable
D51 - Vitamin B12 deficiency anemia	Preventable
D60-D64 - Aplastic and other anemias and other bone marrow failure syndromes	Non-preventable
D61 - Other aplastic anemias and other bone marrow failure syndromes	Non-preventable
D63 - Anemia in chronic diseases classified elsewhere	Non-preventable
D64 - Other anemias	Preventable
D65-D69 - Coagulation defects, purpura and other hemorrhagic conditions	Non-preventable
D69 - Purpura and other hemorrhagic conditions	Non-preventable
Dengue fever [classical dengue] (A90)	Non-preventable
Dengue hemorrhagic fever (A91)	Non-preventable
Dermatopolymyositis (M33)	Non-preventable
Diabetes mellitus due to underlying condition (E08)	Preventable
Diseases of vocal cords and larynx, not elsewhere classified (J38)	Non-preventable
Dislocation and sprain of joints and ligaments at ankle, foot and toe level (S93)	Non-preventable
Disloc and sprain of joints and ligaments of shoulder girdle (S43)	Non-preventable
Dislocation and sprain of joints and ligaments of elbow (S53)	Non-preventable
Dislocation and sprain of joints and ligaments of knee (S83)	Non-preventable
Disorder of continuity of bone (M84)	Non-preventable
Disorders of globe (H44)	Non-preventable
Disorders of lipoprotein metabolism and other lipidemias (E78)	Preventable
Disorders of prepuce (N47)	Non-preventable
Disorders of trigeminal nerve (G50)	Non-preventable
Disorders of vestibular function (H81)	Non-preventable
Disorders of vitreous body (H43)	Non-preventable
Dizziness and giddiness (R42)	Preventable
Dorsalgia (M54)	Preventable
Drug or chemical induced diabetes mellitus (E09)	Preventable
E00-E07 - Disorders of thyroid gland	Preventable
E00-E89 - Endocrine, nutritional and metabolic diseases	Preventable
E03 - Other hypothyroidism	Preventable
E08 - Diabetes mellitus due to underlying condition	Preventable
E08-E13 - Diabetes mellitus	Preventable
E09 – Drug- or chemical -induced diabetes mellitus	Non-preventable
E11 - Type 2 diabetes mellitus	Preventable
E13 - Other specified diabetes mellitus	Preventable
E15-E16 - Other disorders of glucose regulation and pancreatic internal secretion	Non-preventable
E16 - Other disorders of pancreatic internal secretion	Preventable
E23 - Hypofunction and other disorders of the pituitary gland	Non-preventable
E28 - Ovarian dysfunction	Non-preventable
E84 - Cystic fibrosis	Non-preventable
E86 - Volume depletion	Non-preventable
E87 - Other disorders of fluid, electrolyte and acid-base balance	Non-preventable
E89 - Postprocedural endocrine and metabolic complications and disorders, not elsewhere classified	Non-preventable
Ectopic pregnancy (O00)	Non-preventable
Effects of heat and light (T67)	Preventable
Elevated blood glucose level (R73)	Preventable
Emergency use of U07 (U07)	Non-preventable
Encounter for general exam w/o complaint, susp or reported diagnosis (Z00)	Non-preventable
Encounter for adjustment and management of implanted device (Z45)	Non-preventable
Encounter for care involving renal dialysis (Z49)	Non-preventable
Encounter for fitting and adjustment of other devices (Z46)	Non-preventable
Endometriosis (N80)	Non-preventable
Enthesopathies, lower limb, excluding foot (M76)	Non-preventable
Epilepsy and recurrent seizures (G40)	Non-preventable
Essential (primary) hypertension (I10)	Preventable
Excessive, frequent and irregular menstruation (N92)	Preventable
F06 - Other mental disorders due to known physiological condition	Non-preventable
Facial nerve disorders (G51)	Non-preventable
Femoral hernia (K41)	Non-preventable
Fever of other and unknown origin (R50)	Non-preventable
Fissure and fistula of anal and rectal regions (K60)	Preventable
Follicular cysts of skin and subcutaneous tissue (L72)	Non-preventable
Fracture at wrist and hand level (S62)	Non-preventable
Fracture of cervical vertebra and other parts of neck (S12)	Non-preventable
Fracture of femur (S72)	Non-preventable
Fracture of foot and toe, except ankle (S92)	Non-preventable
Fracture of forearm (S52)	Non-preventable
Fracture of lower leg, including ankle (S82)	Non-preventable
Fracture of lumbar spine and pelvis (S32)	Non-preventable
Fracture of rib(s), sternum and thoracic spine (S22)	Non-preventable
Fracture of shoulder and upper arm (S42)	Non-preventable
Fracture of skull and facial bones (S02)	Non-preventable
G03 - Meningitis due to other and unspecified causes	Non-preventable
G04 - Encephalitis, myelitis and encephalomyelitis	Non-preventable
G31 - Other degenerative diseases of nervous system, not elsewhere classified	Non-preventable
G40 - Epilepsy and recurrent seizures	Non-preventable
G40-G47 - Episodic and paroxysmal disorders	Non-preventable
G43 - Migraine	Non-preventable
G45 - Transient cerebral ischemic attacks and related syndromes	Preventable
G459 - Transient cerebral ischemic attack, unspecified	Non-preventable
G46 - Vascular syndromes of brain in cerebrovascular diseases	Preventable
G50-G59 - Nerve, nerve root and plexus disorders	Non-preventable
G52 - Disorders of other cranial nerves	Non-preventable
G56 - Mononeuropathies of upper limb	Non-preventable
G560 - Carpal tunnel syndrome	Non-preventable
G5603 - Carpal tunnel syndrome, bilateral upper limbs	Non-preventable
G57 - Mononeuropathies of lower limb	Preventable
G60-G65 - Polyneuropathies and other disorders of the peripheral nervous system	Non-preventable
G61 - Inflammatory polyneuropathy	Non-preventable
G80-G83 - Cerebral palsy and other paralytic syndromes	Non-preventable
G81 - Hemiplegia and hemiparesis	Non-preventable
G89-G99 - Other disorders of the nervous system	Preventable
G93 - Other disorders of brain	Non-preventable
G97 - Intraoperative and postprocedural complications and disorders of nervous system, not elsewhere classified	Non-preventable
Gangrene, not elsewhere classified (I96)	Preventable
Gastritis and duodenitis (K29)	Preventable
Glaucoma (H40)	Non-preventable
Granulomatous disorders of skin and subcutaneous tissue (L92)	Non-preventable
H00 - Hordeolum and chalazion	Non-preventable
H00-H05 - Disorders of eyelid, lacrimal system and orbit	Non-preventable
H00-H59 - Diseases of the eye and adnexa	Non-preventable
H01 - Other inflammation of eyelid	Non-preventable
H02 - Other disorders of eyelid	Non-preventable
H04 - Disorders of lacrimal system	Non-preventable
H040 - Dacryoadenitis	Non-preventable
H05 - Disorders of orbit	Non-preventable
H10 - Conjunctivitis	Non-preventable
H11 - Other disorders of conjunctiva	Non-preventable
H18 - Other disorders of cornea	Non-preventable
H25 - Age-related cataract	Non-preventable
H25-H28 - Disorders of lens	Non-preventable
H2501 - Cortical age-related cataract	Non-preventable
H25011 - Cortical age-related cataract, right eye	Non-preventable
H25012 - Cortical age-related cataract, left eye	Non-preventable
H25013 - Cortical age-related cataract, bilateral	Non-preventable
H2511 - Age-related nuclear cataract, right eye	Non-preventable
H2511 - Cortical age-related cataract, right eye	Non-preventable
H2512 - Age-related nuclear cataract, left eye	Non-preventable
H252 - Age-related cataract, morgagnian type	Non-preventable
H25812 - Combined forms of age-related cataract, left eye	Non-preventable
H259 - Unspecified age-related cataract	Non-preventable
H26 - Other cataract	Non-preventable
H269 - Unspecified cataract	Non-preventable
H30 - Chorioretinal inflammation	Non-preventable
H30-H36 - Disorders of choroid and retina	Non-preventable
H31 - Other disorders of choroid	Non-preventable
H33 - Retinal detachments and breaks	Non-preventable
H330 - Retinal detachment with retinal break	Non-preventable
H34 - Retinal vascular occlusions	Non-preventable
H348120 - Central retinal vein occlusion, left eye, with macular edema	Non-preventable
H35 - Other retinal disorders	Non-preventable
H40 - Glaucoma	Non-preventable
H40-H42 - Glaucoma	Non-preventable
H43 - Disorders of vitreous body	Non-preventable
H43-H44 - Disorders of vitreous body and globe	Non-preventable
H44 - Disorders of globe	Non-preventable
H46 - Optic neuritis	Non-preventable
H50 - Other strabismus	Non-preventable
H53 - Visual disturbances	Non-preventable
H53-H54 - Visual disturbances and blindness	Non-preventable
H54 - Blindness and low vision	Non-preventable
H55-H57 - Other disorders of eye and adnexa	Non-preventable
H57 - Other disorders of eye and adnexa	Non-preventable
H59 - Intraoperative and postprocedural complications and disorders of eye and adnexa, not elsewhere classified	Non-preventable
H60 - Otitis externa	Non-preventable
H6090 - Unspecified otitis externa, unspecified ear	Non-preventable
H65 - Nonsuppurative otitis media	Non-preventable
H65-H75 - Diseases of middle ear and mastoid	Non-preventable
H66 - Suppurative and unspecified otitis media	Non-preventable
H72 - Perforation of tympanic membrane	Non-preventable
H74 - Other disorders of middle ear mastoid	Non-preventable
H80 - Otosclerosis	Non-preventable
H81 - Disorders of vestibular function	Non-preventable
H83 - Other diseases of inner ear	Non-preventable
H90 - Conductive and sensorineural hearing loss	Non-preventable
H90-H94 - Other disorders of ear	Non-preventable
H91 - Other and unspecified hearing loss	Non-preventable
H92 - Otalgia and effusion of ear	Non-preventable
Headache (R51)	Preventable
Heart failure (I50)	Preventable
Hemangioma and lymphangioma, any site (D18)	Non-preventable
Hematuria (R31)	Non-preventable
Hemorrhage from respiratory passages (R04)	Non-preventable
Hemorrhoids and perianal venous thrombosis (K64)	Preventable
Hepatic failure, not elsewhere classified (K72)	Non-preventable
Herpesviral [herpes simplex] infections (B00)	Non-preventable
Hydrocele and spermatocele - Hydrocele and spermatocele	Non-preventable
Hydrocele and spermatocele (N43)	Non-preventable
Hypertensive crisis (I16)	Preventable
Hypertensive heart and chronic kidney disease (I13)	Preventable
Hypertensive heart disease (I11)	Preventable
I10 - Essential (primary) hypertension	Preventable
I10-I16 - Hypertensive diseases	Preventable
I11 - Hypertensive heart disease	Preventable
I15 - Secondary hypertension	Preventable
I16 - Hypertensive crisis	Preventable
I20 - Angina pectoris	Non-preventable
I20-I25 - Ischemic heart diseases	Preventable
I200 - Unstable angina	Non-preventable
I21 - ST elevation (STEMI) and non-ST elevation (NSTEMI) myocardial infarction	Preventable
I214 - Non-ST elevation (NSTEMI) myocardial infarction	Preventable
I23 - Certain current complications following ST elevation (STEMI) and non-ST elevation (NSTEMI) myocardial infarction (within the 28 -day period)	Non-preventable
I24 - Other acute ischemic heart diseases	Preventable
I249 - Acute ischemic heart disease, unspecified	Preventable
I25 - Chronic ischemic heart disease	Preventable
I251 - Atherosclerotic heart disease of native coronary artery	Preventable
I2510 - Atherosclerotic heart disease of native coronary artery without angina pectoris	Preventable
I25810 - Atherosclerosis of coronary artery bypass graft(s) without angina pectoris	Preventable
I26 - Pulmonary embolism	Non-preventable
I26-I28 - Pulmonary heart disease and diseases of pulmonary circulation	Preventable
I30-I52 - Other forms of heart disease	Preventable
I31 - Other diseases of pericardium	Non-preventable
I35 - Nonrheumatic aortic valve disorders	Preventable
I42 - Cardiomyopathy	Preventable
I44 - Atrioventricular and left bundle-branch block	Preventable
I45 - Other conduction disorders	Preventable
I47 - Paroxysmal tachycardia	Preventable
I48 - Atrial fibrillation and flutter	Preventable
I49 - Other cardiac arrhythmias	Non-preventable
I50 - Heart failure	Preventable
I60-I69 - Cerebrovascular diseases	Preventable
I61 - Nontraumatic intracerebral hemorrhage	Preventable
I62 - Other and unspecified nontraumatic intracranial hemorrhage	Non-preventable
I63 - Cerebral infarction	Preventable
I639 - Cerebral infarction, unspecified	Preventable
I67 - Other cerebrovascular diseases	Non-preventable
I69 - Sequelae of cerebrovascular disease	Non-preventable
I69351 - Hemiplegia and hemiparesis following cerebral infarction affecting right dominant side	Non-preventable
I70 - Atherosclerosis	Preventable
I70-I79 - Diseases of arteries, arterioles and capillaries	Preventable
I72 - Other aneurysm	Non-preventable
I77 - Other disorders of arteries and arterioles	Non-preventable
I80-I89 - Diseases of veins, lymphatic vessels and lymph nodes, not elsewhere classified	Non-preventable
I82 - Other venous embolism and thrombosis	Preventable
I83 - Varicose veins of lower extremities	Preventable
I83813 - Varicose veins of bilateral lower extremities with pain	Preventable
I87 - Other disorders of veins	Non-preventable
Illness, unspecified (R69)	Non-preventable
Infectious gastroenteritis and colitis, unspecified (A09)	Non-preventable
Infectious mononucleosis (B27)	Non-preventable
Inflammatory diseases of prostate (N41)	Non-preventable
Inflammatory disorders of breast (N61)	Non-preventable
Inflammatory disorders of male genital organs, not elsewhere classified (N49)	Non-preventable
Inflammatory polyneuropathy (G61)	Non-preventable
Influenza due to certain identified influenza viruses (J09)	Non-preventable
Influenza due to other identified influenza virus (J10)	Non-preventable
Influenza due to unidentified influenza virus (J11)	Non-preventable
Inguinal hernia (K40)	Non-preventable
Injury of nerves and lumbar spinal cord at abdomen, lower back and pelvis level (S34)	Non-preventable
Injury of blood vessels at wrist and hand level (S65)	Non-preventable
Injury of eye and orbit (S05)	Non-preventable
Injury of intra-abdominal organs (S36)	Non-preventable
Injury of nerves at shoulder and upper arm level (S44)	Non-preventable
Injury of other and unspecified intrathoracic organs (S27)	Non-preventable
Internal derangement of knee (M23)	Non-preventable
Intracranial and intraspinal abscess and granuloma (G06)	Non-preventable
Intracranial injury (S06)	Non-preventable
Intraoperative and postprocedural complications and disorders of digestive system, not elsewhere classified (K91)	Non-preventable
Intraoperative and postprocedural complications and disorders of genitourinary system, not elsewhere classified (N99)	Non-preventable
Intraoperative and postprocedural complications and disorders of musculoskeletal system, not elsewhere classified (M96)	Non-preventable
Intraoperative and postprocedural complications and disorders of nervous system, not elsewhere classified (G97)	Non-preventable
Iron deficiency anemia (D50)	Preventable
IVDP - IVDP	Non-preventable
J00-J06 - Acute upper respiratory infections	Non-preventable
J00-J99 - Diseases of the respiratory system	Non-preventable
J01 - Acute sinusitis	Preventable
J03 - Acute tonsillitis	Non-preventable
J0390 - Acute tonsillitis, unspecified	Non-preventable
J06 - Acute upper respiratory infections of multiple and unspecified sites	Non-preventable
J09-J18 - Influenza and pneumonia	Non-preventable
J10 - Influenza due to other identified influenza virus	Non-preventable
J11 - Influenza due to unidentified influenza virus	Non-preventable
J12 - Viral pneumonia, not elsewhere classified	Non-preventable
J18 - Pneumonia, unspecified organism	Non-preventable
J20 - Acute bronchitis	Non-preventable
J20-J22 - Other acute lower respiratory infections	Non-preventable
J30-J39 - Other diseases of upper respiratory tract	Non-preventable
J32 - Chronic sinusitis	Preventable
J33 - Nasal polyp	Non-preventable
J34 - Other and unspecified disorders of nose and nasal sinuses	Non-preventable
J342 - Deviated nasal septum	Non-preventable
J35 - Chronic diseases of tonsils and adenoids	Non-preventable
J38 - Diseases of vocal cords and larynx, not elsewhere classified	Non-preventable
J40-J47 - Chronic lower respiratory diseases	Non-preventable
J44 - Other chronic obstructive pulmonary disease	Non-preventable
J45 - Asthma	Preventable
J479 - Bronchiectasis, uncomplicated	Non-preventable
J69 - Pneumonitis due to solids and liquids	Non-preventable
J80-J84 - Other respiratory diseases principally affecting the interstitium	Non-preventable
J84 - Other interstitial pulmonary diseases	Non-preventable
J86 - Pyothorax	Non-preventable
J90-J94 - Other diseases of the pleura	Non-preventable
J91 - Pleural effusion in conditions classified elsewhere	Non-preventable
J93 - Pneumothorax and air leak	Non-preventable
J94 - Other pleural conditions	Non-preventable
J95 - Intraoperative and postprocedural complications and disorders of respiratory system, not elsewhere classified	Non-preventable
J95830 - Postprocedural hemorrhage of a respiratory system organ or structure following a respiratory system procedure	Non-preventable
J96 - Respiratory failure, not elsewhere classified	Non-preventable
J96-J99 - Other diseases of the respiratory system	Non-preventable
J98 - Other respiratory disorders	Non-preventable
K00-K14 - Diseases of oral cavity and salivary glands	Non-preventable
K00-K95 - Diseases of the digestive system	Non-preventable
K11 - Diseases of salivary glands	Non-preventable
K12 - Stomatitis and related lesions	Non-preventable
K13 - Other diseases of lip and oral mucosa	Non-preventable
K20-K31 - Diseases of esophagus, stomach and duodenum	Non-preventable
K28 - Gastrojejunal ulcer	Non-preventable
K29 - Gastritis and duodenitis	Non-preventable
K31 - Other diseases of stomach and duodenum	Non-preventable
K35 - Acute appendicitis	Non-preventable
K35-K38 - Diseases of appendix	Non-preventable
K40 - Inguinal hernia	Non-preventable
K40-K46 - Hernia	Non-preventable
K42 - Umbilical hernia	Non-preventable
K43 - Ventral hernia	Non-preventable
K46 - Unspecified abdominal hernia	Non-preventable
K469 - Unspecified abdominal hernia without obstruction or gangrene	Non-preventable
K50-K52 - Noninfective enteritis and colitis	Non-preventable
K51 - Ulcerative colitis	Preventable
K52 - Other and unspecified noninfective gastroenteritis and colitis	Non-preventable
K529 - Noninfective gastroenteritis and colitis, unspecified	Non-preventable
K55-K64 - Other diseases of intestines	Non-preventable
K57 - Diverticular disease of intestine	Preventable
K60 - Fissure and fistula of anal and rectal regions	Non-preventable
K602 - Anal fissure, unspecified	Preventable
K603 - Anal fistula	Non-preventable
K61 - Abscess of anal and rectal regions	Non-preventable
K62 - Other diseases of anus and rectum	Preventable
K63 - Other diseases of intestine	Non-preventable
K64 - Hemorrhoids and perianal venous thrombosis	Preventable
K649 - Unspecified hemorrhoids	Preventable
K70-K77 - Diseases of liver	Non-preventable
K74 - Fibrosis and cirrhosis of liver	Preventable
K75 - Other inflammatory liver diseases	Non-preventable
K76 - Other diseases of liver	Preventable
K80 - Cholelithiasis	Non-preventable
K80-K87 - Disorders of gallbladder, biliary tract and pancreas	Non-preventable
K8000 - Calculus of gallbladder with acute cholecystitis without obstruction	Non-preventable
K81 - Cholecystitis	Non-preventable
K83 - Other diseases of biliary tract	Non-preventable
K85 - Acute pancreatitis	Non-preventable
K86 - Other diseases of pancreas	Non-preventable
K90-K95 - Other diseases of the digestive system	Non-preventable
K91 - Intraoperative and postprocedural complications and disorders of digestive system, not elsewhere classified	Non-preventable
K92 - Other diseases of digestive system	Non-preventable
Keratitis (H16)	Non-preventable
L00-L08 - Infections of the skin and subcutaneous tissue	Non-preventable
L02 - Cutaneous abscess, furuncle and carbuncle	Non-preventable
L03 - Cellulitis and acute lymphangitis	Non-preventable
L05 - Pilonidal cyst and sinus	Non-preventable
L08 - Other local infections of skin and subcutaneous tissue	Non-preventable
L40 - Psoriasis	Non-preventable
L49-L54 - Urticaria and erythema	Non-preventable
L60-L75 - Disorders of skin appendages	Non-preventable
L72 - Follicular cysts of skin and subcutaneous tissue	Non-preventable
L720 - Epidermal cyst	Non-preventable
L73 - Other follicular disorders	Non-preventable
L80-L99 - Other disorders of the skin and subcutaneous tissue	Non-preventable
L89 - Pressure ulcer	Preventable
L97 - Non-pressure chronic ulcer of lower limb, not elsewhere classified	Non-preventable
Leiomyoma of uterus (D25)	Non-preventable
Localized swelling, mass and lump of skin and subcutaneous tissue (R22)	Non-preventable
M00 - Pyogenic arthritis	Non-preventable
M00-M99 - Diseases of the musculoskeletal system and connective tissue	Non-preventable
M02 - Postinfective and reactive arthropathies	Non-preventable
M05 - Rheumatoid arthritis with rheumatoid factor	Non-preventable
M05-M14 - Inflammatory polyarthropathies	Non-preventable
M12 - Other and unspecified arthropathy	Non-preventable
M15 - Polyosteoarthritis	Non-preventable
M15-M19 - Osteoarthritis	Non-preventable
M16 - Osteoarthritis of hip	Non-preventable
M17 - Osteoarthritis of knee	Non-preventable
M170 - Bilateral primary osteoarthritis of knee	Non-preventable
M189 - Osteoarthritis of first carpometacarpal joint, unspecified	Non-preventable
M19 - Other and unspecified osteoarthritis	Preventable
M20 - Acquired deformities of fingers and toes	Non-preventable
M20-M25 - Other joint disorders	Non-preventable
M21 - Other acquired deformities of limbs	Non-preventable
M22 - Disorder of patella	Non-preventable
M23 - Internal derangement of knee	Non-preventable
M24 - Other specific joint derangements	Non-preventable
M25 - Other joint disorder, not elsewhere classified	Preventable
M30-M36 - Systemic connective tissue disorders	Non-preventable
M31 - Other necrotizing vasculopathies	Non-preventable
M32 - Systemic lupus erythematosus (SLE)	Non-preventable
M43 - Other deforming dorsopathies	Non-preventable
M45-M49 - Spondylopathies	Preventable
M46 - Other inflammatory spondylopathies	Non-preventable
M47 - Spondylosis	Preventable
M48 - Other spondylopathies	Non-preventable
M50 - Cervical disc disorders	Non-preventable
M50-M54 - Other dorsopathies	Non-preventable
M51 - Thoracic, thoracolumbar, and lumbosacral intervertebral disc disorders	Non-preventable
M53 - Other and unspecified dorsopathies, not elsewhere classified	Non-preventable
M54 - Dorsalgia	Preventable
M62 - Other disorders of muscle	Non-preventable
M6208 - Separation of muscle (nontraumatic), other site	Non-preventable
M65 - Synovitis and tenosynovitis	Non-preventable
M65-M67 - Disorders of synovium and tendon	Non-preventable
M65311 - Trigger thumb, right thumb	Non-preventable
M654 - Radial styloid tenosynovitis [de Quervain]	Non-preventable
M66 - Spontaneous rupture of synovium and tendon	Non-preventable
M67 - Other disorders of synovium and tendon	Non-preventable
M70-M79 - Other soft tissue disorders	Non-preventable
M75 - Shoulder lesions	Non-preventable
M75122 - Complete rotator cuff tear or rupture of left shoulder, not specified as traumatic	Non-preventable
M79 - Other and unspecified soft tissue disorders, not elsewhere classified	Non-preventable
M80 - Osteoporosis with current pathological fracture	Non-preventable
M80-M85 - Disorders of bone density and structure	Non-preventable
M84 - Disorder of continuity of bone	Non-preventable
M85 - Other disorders of bone density and structure	Non-preventable
M87 - Osteonecrosis	Non-preventable
M8708 - Idiopathic aseptic necrosis of bone, other site	Non-preventable
M94 - Other disorders of cartilage	Non-preventable
M96 - Intraoperative and postprocedural complications and disorders of musculoskeletal system, not elsewhere classified	Non-preventable
Malignant immunoproliferative diseases and certain other B-cell lymphomas (C88)	Non-preventable
Malignant melanoma of skin (C43)	Non-preventable
Malignant neoplasm of bone and articular cartilage of other and unspecified sites (C41)	Non-preventable
Malignant neoplasm of breast (C50)	Non-preventable
Malignant neoplasm of bronchus and lung (C34)	Non-preventable
Malignant neoplasm of cervix uteri (C53)	Non-preventable
Malignant neoplasm of corpus uteri (C54)	Non-preventable
Malignant neoplasm of endo glands and related structures (C75)	Non-preventable
Malignant neoplasm of esophagus (C15)	Non-preventable
Malignant neoplasm of hypopharynx (C13)	Non-preventable
Malignant neoplasm of kidney, except renal pelvis (C64)	Non-preventable
Malignant neoplasm of larynx (C32)	Non-preventable
Malignant neoplasm of oropharynx (C10)	Non-preventable
Malignant neoplasm of other and ill-defined sites (C76)	Non-preventable
Malignant neoplasm of other and unspecified female genital organs (C57)	Non-preventable
Malignant neoplasm of other and unspecified parts of biliary tract (C24)	Non-preventable
Malignant neoplasm of other and unspecified parts of mouth (C06)	Non-preventable
Malignant neoplasm of other and unspecified parts of tongue (C02)	Non-preventable
Malignant neoplasm of ovary (C56)	Non-preventable
Malignant neoplasm of parotid gland (C07)	Non-preventable
Malignant neoplasm of prostate (C61)	Non-preventable
Malignant neoplasm of rectum (C20)	Non-preventable
Malignant neoplasm of stomach (C16)	Non-preventable
Malignant neoplasm without specification of site (C80)	Non-preventable
Menopausal and other perimenopausal disorders (N95)	Preventable
Merkel cell carcinoma (C4A)	Non-preventable
Mononeuropathies of upper limb (G56)	Non-preventable
Motor- or nonmotor-vehicle accident, type of vehicle unspecified (V89)	Non-preventable
Mumps (B26)	Non-preventable
Myositis (M60)	Non-preventable
N00-N08 - Glomerular diseases	Non-preventable
N04 - Nephrotic syndrome	Non-preventable
N10 - Acute pyelonephritis	Preventable
N10-N16 - Renal tubulo-interstitial diseases	Preventable
N11 - Chronic tubulo-interstitial nephritis	Non-preventable
N13 - Obstructive and reflux uropathy	Non-preventable
N17 - Acute kidney failure	Non-preventable
N17-N19 - Acute kidney failure and chronic kidney disease	Non-preventable
N18 - Chronic kidney disease (CKD)	Non-preventable
N189 - Chronic kidney disease, unspecified	Non-preventable
N20 - Calculus of kidney and ureter	Non-preventable
N20-N23 - Urolithiasis	Non-preventable
N200 - Calculus of kidney	Non-preventable
N201 - Calculus of ureter	Non-preventable
N209 - Urinary calculus, unspecified	Non-preventable
N21 - Calculus of lower urinary tract	Non-preventable
N25-N29 - Other disorders of kidney and ureter	Non-preventable
N28 - Other disorders of kidney and ureter, not elsewhere classified	Non-preventable
N30 - Cystitis	Non-preventable
N30-N39 - Other diseases of the urinary system	Non-preventable
N32 - Other disorders of bladder	Non-preventable
N35 - Urethral stricture	Non-preventable
N359 - Urethral stricture, unspecified	Non-preventable
N39 - Other disorders of urinary system	Preventable
N390 - Urinary tract infection, site not specified	Preventable
N40 - Benign prostatic hyperplasia	Non-preventable
N40-N53 - Diseases of male genital organs	Non-preventable
N41 - Inflammatory diseases of prostate	Non-preventable
N43 - Hydrocele and spermatocele	Non-preventable
N45 - Orchitis and epididymitis	Non-preventable
N47 - Disorders of prepuce	Non-preventable
N471 - Phimosis	Non-preventable
N48 - Other disorders of penis	Non-preventable
N49 - Inflammatory disorders of male genital organs, not elsewhere classified	Non-preventable
N60 - Benign mammary dysplasia	Non-preventable
N60-N65 - Disorders of breast	Non-preventable
N61 - Inflammatory disorders of breast	Non-preventable
N63 - Unspecified lump in breast	Non-preventable
N70-N77 - Inflammatory diseases of female pelvic organs	Preventable
N71 - Inflammatory disease of uterus, except cervix	Non-preventable
N76 - Other inflammation of vagina and vulva	Non-preventable
N80 - Endometriosis	Non-preventable
N80-N98 - Noninflammatory disorders of female genital tract	Non-preventable
N81 - Female genital prolapse	Non-preventable
N83 - Noninflammatory disorders of ovary, fallopian tube and broad ligament	Non-preventable
N83291 - Other ovarian cyst, right side	Non-preventable
N84 - Polyp of female genital tract	Non-preventable
N85 - Other noninflammatory disorders of uterus, except cervix	Non-preventable
N92 - Excessive, frequent and irregular menstruation	Preventable
N93 - Other abnormal uterine and vaginal bleeding	Non-preventable
N939 - Abnormal uterine and vaginal bleeding, unspecified	Non-preventable
N95 - Menopausal and other perimenopausal disorders	Non-preventable
N99 - Intraoperative and postprocedural complications and disorders of genitourinary system, not elsewhere classified	Non-preventable
Nail disorders (L60)	Non-preventable
Nasal polyp (J33)	Non-preventable
Nausea and vomiting (R11)	Non-preventable
Neoplasm of uncertain behavior of male genital organs (D40)	Non-preventable
Neoplasm of uncertain behavior of oral cavity and digestive organs (D37)	Non-preventable
Nephrotic syndrome (N04)	Non-preventable
Neuromuscular dysfunction of bladder, not elsewhere classified (N31)	Non-preventable
Neutropenia (D70)	Non-preventable
Noninflammatory disorders of ovary, fallopian tube and broad ligament (N83)	Non-preventable
Noninflammatory disorders of testis (N44)	Non-preventable
Nonsuppurative otitis media (H65)	Non-preventable
Nontraffic accident of specified type but victim's mode of transport unknown (V88)	Non-preventable
Nontraumatic intracerebral hemorrhage (I61)	Non-preventable
Nontraumatic subarachnoid hemorrhage (I60)	Non-preventable
O00 - Ectopic pregnancy	Non-preventable
Obstructive and reflux uropathy (N13)	Non-preventable
Occupant of special all-terrain or other off-road motor vehicle, injured in transport accident (V86)	Non-preventable
Open wound of ankle, foot and toes (S91)	Non-preventable
Orchitis and epididymitis (N45)	Non-preventable
Osteoarthritis of knee (M17)	Preventable
Osteonecrosis (M87)	Non-preventable
Osteoporosis with current pathological fracture (M80)	Non-preventable
Oth aplastic anemias and other bone marrow failure syndromes (D61)	Non-preventable
Other disorders of skin and subcutaneous tissue, not elsewhere classified (L98)	Non-preventable
Oth symptoms and signs involving the digestive sys and abdomen (R19)	Preventable
Oth transitory neonatal electrolyte and metabolic disturb (P74)	Non-preventable
Other abnormal uterine and vaginal bleeding (N93)	Non-preventable
Other acquired deformities of limbs (M21)	Non-preventable
Other acute ischemic heart diseases (I24)	Preventable
Other acute viral hepatitis (B17)	Non-preventable
Other and unspecified noninfective gastroenteritis and colitis (K52)	Non-preventable
Other and unspecified arthropathy (M12)	Non-preventable
Other and unspecified disorders of nose and nasal sinuses (J34)	Non-preventable
Other and unspecified dorsopathies, not elsewhere classified (M53)	Non-preventable
Other and unspecified injuries of ankle and foot (S99)	Non-preventable
Other and unspecified injuries of elbow and forearm (S59)	Non-preventable
Other and unspecified injuries of head (S09)	Non-preventable
Other and unspecified malignant neoplasm of skin (C44)	Non-preventable
Other and unspecified nontraumatic intracranial hemorrhage (I62)	Non-preventable
Other and unspecified osteoarthritis (M19)	Preventable
Other arthropod-borne viral fevers, not elsewhere classified (A93)	Non-preventable
Other bacterial intestinal infections (A04)	Non-preventable
Other benign neoplasms of connective and other soft tissue (D21)	Non-preventable
Other bursopathies (M71)	Non-preventable
Other cataract (H26)	Non-preventable
Other cerebrovascular diseases (I67)	Non-preventable
Other chronic obstructive pulmonary disease (J44)	Non-preventable
Other conduction disorders (I45)	Non-preventable
Other crystal arthropathies (M11)	Non-preventable
Other deforming dorsopathies (M43)	Preventable
Other diseases of appendix (K38)	Non-preventable
Other diseases of digestive system (K92)	Non-preventable
Other diseases of gallbladder (K82)	Non-preventable
Other diseases of lip and oral mucosa (K13)	Non-preventable
Other diseases of liver (K76)	Preventable
Other diseases of pancreas (K86)	Non-preventable
Other diseases of stomach and duodenum (K31)	Non-preventable
Other diseases of upper respiratory tract (J39)	Non-preventable
Other disorders of arteries and arterioles (I77)	Non-preventable
Other disorders of bladder (N32)	Non-preventable
Other disorders of bone (M89)	Non-preventable
Other disorders of brain (G93)	Non-preventable
Other disorders of conjunctiva (H11)	Non-preventable
Other disorders of cornea (H18)	Non-preventable
Other disorders of eyelid (H02)	Non-preventable
Other disorders of fluid, electrolyte and acid-base balance (E87)	Non-preventable
Other disorders of lens (H27)	Non-preventable
Other disorders of synovium and tendon (M67)	Non-preventable
Other disorders of urinary system (N39)	Preventable
Other headache syndromes (G44)	Non-preventable
Other inflammatory liver diseases (K75)	Preventable
Other inflammatory spondylopathies (M46)	Non-preventable
Other interstitial pulmonary diseases (J84)	Non-preventable
Other joint disorder, not elsewhere classified (M25)	Non-preventable
Other lack of coordination (R27)	Non-preventable
Other mosquito-borne viral fevers (A92)	Non-preventable
Other noninflammatory disorders of cervix uteri (N88)	Non-preventable
Other noninflammatory disorders of uterus, except cervix (N85)	Non-preventable
Other postprocedural states (Z98)	Non-preventable
Other pulmonary heart diseases (I27)	Preventable
Other respiratory disorders (J98)	Non-preventable
Other retinal disorders (H35)	Non-preventable
Other salmonella infections (A02)	Non-preventable
Other sepsis (A41)	Non-preventable
Other specific joint derangements (M24)	Non-preventable
Other specified diabetes mellitus (E13)	Preventable
Other specified malaria (B53)	Non-preventable
Other spondylopathies (M48)	Non-preventable
Otitis externa (H60)	Non-preventable
P91 - Other disturbances of cerebral status of newborn	Non-preventable
Pain and other condition associated with female genital organs and menstrual cycle (N94)	Preventable
Pain in throat and chest (R07)	Preventable
Pain, not elsewhere classified (G89)	Preventable
Paralytic ileus and intestinal obstruction without hernia (K56)	Non-preventable
Parkinson's disease (G20)	Non-preventable
Paroxysmal tachycardia (I47)	Preventable
Pedestrian injured in collision with heavy transport vehicle (V04)	Non-preventable
Pedestrian injured in other and unspecified transport accidents (V09)	Non-preventable
Periprosthetic fracture around internal prosthetic joint (M97)	Non-preventable
Peritonitis (K65)	Non-preventable
Personal history of malignant neoplasm (Z85)	Non-preventable
Personal history of medical treatment (Z92)	Non-preventable
Personal history of other diseases and conditions (Z87)	Non-preventable
Pilonidal cyst and sinus (L05)	Non-preventable
Plasmodium falciparum malaria (B50)	Non-preventable
Plasmodium malariae malaria (B52)	Non-preventable
Plasmodium vivax malaria (B51)	Non-preventable
Pleural effusion in conditions classified elsewhere (J91)	Non-preventable
Pneumonia, unspecified organism (J18)	Non-preventable
Pneumonitis due to solids and liquids (J69)	Non-preventable
Polyosteoarthritis (M15)	Preventable
Polyp of female genital tract (N84)	Non-preventable
Portal vein thrombosis (I81)	Non-preventable
Presence of other functional implants (Z96)	Non-preventable
Purpura and other hemorrhagic conditions (D69)	Non-preventable
Q40 - Other congenital malformations of upper alimentary tract	Non-preventable
Q44 - Congenital malformations of gallbladder, bile ducts and liver	Non-preventable
Q64 - Other congenital malformations of urinary system	Non-preventable
R00-R09 - Symptoms and signs involving the circulatory and respiratory systems	Preventable
R03 - Abnormal blood-pressure reading, without diagnosis	Preventable
R04 - Hemorrhage from respiratory passages	Non-preventable
R06 - Abnormalities of breathing	Preventable
R0602 - Shortness of breath	Non-preventable
R07 - Pain in throat and chest	Preventable
R079 - Chest pain, unspecified	Preventable
R10 - Abdominal and pelvic pain	Preventable
R10-R19 - Symptoms and signs involving the digestive system and abdomen	Non-preventable
R11 - Nausea and vomiting	Non-preventable
R1314 - Dysphagia, pharyngoesophageal phase	Non-preventable
R19 - Other symptoms and signs involving the digestive system and abdomen	Preventable
R20 - Disturbances of skin sensation	Non-preventable
R20-R23 - Symptoms and signs involving the skin and subcutaneous tissue	Non-preventable
R22 - Localized swelling, mass and lump of skin and subcutaneous tissue	Non-preventable
R222 - Localized swelling, mass and lump, trunk	Non-preventable
R29 - Other symptoms and signs involving the nervous and musculoskeletal systems	Non-preventable
R50 - Fever of other and unknown origin	Non-preventable
R50-R69 - General symptoms and signs	Non-preventable
R5081 - Fever presenting with conditions classified elsewhere	Non-preventable
R509 - Fever, unspecified	Non-preventable
R53 - Malaise and fatigue	Preventable
R56 - Convulsions, not elsewhere classified	Non-preventable
R57 - Shock, not elsewhere classified	Non-preventable
R60 - Edema, not elsewhere classified	Non-preventable
R65 - Symptoms and signs specifically associated with systemic inflammation and infection	Non-preventable
R68 - Other general symptoms and signs	Non-preventable
R80 - Proteinuria	Non-preventable
R80-R82 - Abnormal findings on examination of urine, without diagnosis	Non-preventable
Respiratory failure, not elsewhere classified (J96)	Non-preventable
Respiratory tuberculosis (A15)	Non-preventable
Retention of urine (R33)	Non-preventable
Retinal detachments and breaks (H33)	Non-preventable
Retinal vascular occlusions (H34)	Non-preventable
S00 - Superficial injury of head	Non-preventable
S00-S09 - Injuries to the head	Non-preventable
S00-T88 - Injury, poisoning and certain other consequences of external causes	Non-preventable
S01 - Open wound of the head	Non-preventable
S02 - Fracture of skull and facial bones	Non-preventable
S05 - Injury of eye and orbit	Non-preventable
S06 - Intracranial injury	Non-preventable
S09 - Other and unspecified injuries of head	Non-preventable
S20-S29 - Injuries to the thorax	Non-preventable
S22 - Fracture of rib(s), sternum and thoracic spine	Non-preventable
S30 - Superficial injury of abdomen, lower back, pelvis and external genitals	Non-preventable
S30-S39 - Injuries to the abdomen, lower back, lumbar spine, pelvis and external genitals	Non-preventable
S31 - Open wound of abdomen, lower back, pelvis and external genitals	Non-preventable
S32 - Fracture of lumbar spine and pelvis	Non-preventable
S33 - Dislocation and sprain of joints and ligaments of lumbar spine and pelvis	Non-preventable
S35 - Injury of blood vessels at abdomen, lower back and pelvis level	Non-preventable
S36 - Injury of intra-abdominal organs	Non-preventable
S37 - Injury of urinary and pelvic organs	Non-preventable
S40-S49 - Injuries to the shoulder and upper arm	Non-preventable
S42 - Fracture of shoulder and upper arm	Non-preventable
S42201A - Unspecified fracture of upper end of right humerus, initial encounter for closed fracture	Non-preventable
S43 - Dislocation and sprain of joints and ligaments of shoulder girdle	Non-preventable
S46 - Injury of muscle, fascia and tendon at shoulder and upper arm level	Non-preventable
S4601 - Strain of muscle(s) and tendon(s) of the rotator cuff of shoulder	Non-preventable
S49 - Other and unspecified injuries of shoulder and upper arm	Non-preventable
S50-S59 - Injuries to the elbow and forearm	Non-preventable
S52 - Fracture of forearm	Non-preventable
S525 - Fracture of lower end of radius	Non-preventable
S53 - Dislocation and sprain of joints and ligaments of elbow	Non-preventable
S59 - Other and unspecified injuries of elbow and forearm	Non-preventable
S60 - Superficial injury of wrist, hand and fingers	Non-preventable
S60-S69 - Injuries to the wrist, hand and fingers	Non-preventable
S61 - Open wound of wrist, hand and fingers	Non-preventable
S62 - Fracture at wrist and hand level	Non-preventable
S63 - Dislocation and sprain of joints and ligaments at wrist and hand level	Non-preventable
S67 - Crushing injury of wrist, hand and fingers	Non-preventable
S69 - Other and unspecified injuries of wrist, hand and finger(s)	Non-preventable
S70-S79 - Injuries to the hip and thigh	Non-preventable
S72 - Fracture of femur	Non-preventable
S729 - Unspecified fracture of femur	Non-preventable
S7291 - Unspecified fracture of right femur	Non-preventable
S73 - Dislocation and sprain of joint and ligaments of hip	Non-preventable
S80 - Superficial injury of knee and lower leg	Non-preventable
S80-S89 - Injuries to the knee and lower leg	Non-preventable
S82 - Fracture of lower leg, including ankle	Non-preventable
S8212 - Fracture of lateral condyle of tibia	Non-preventable
S83 - Dislocation and sprain of joints and ligaments of knee	Non-preventable
S8353 - Sprain of cruciate ligament of knee	Non-preventable
S86 - Injury of muscle, fascia and tendon at lower leg level	Non-preventable
S90 - Superficial injury of ankle, foot and toes	Non-preventable
S90-S99 - Injuries to the ankle and foot	Non-preventable
S91 - Open wound of ankle, foot and toes	Non-preventable
S92 - Fracture of foot and toe, except ankle	Non-preventable
S93 - Dislocation and sprain of joints and ligaments at ankle, foot and toe level	Non-preventable
S97 - Crushing injury of ankle and foot	Non-preventable
S999 - Unspecified injury of ankle and foot	Non-preventable
Secondary hypertension (I15)	Preventable
Sequelae of cerebrovascular disease (I69)	Preventable
Sleep disorders (G47)	Preventable
Spondylosis (M47)	Preventable
Spontaneous rupture of synovium and tendon (M66)	Non-preventable
Spotted fever [tick-borne rickettsioses] (A77)	Non-preventable
STEMI & NSTEMI myocardial infarction (I21)	Preventable
Stomatitis and related lesions (K12)	Non-preventable
Superficial injury of ankle, foot and toes (S90)	Non-preventable
Superficial injury of head (S00)	Non-preventable
Superficial injury of wrist, hand and fingers (S60)	Non-preventable
Suppurative and unspecified otitis media (H66)	Non-preventable
Syncope and collapse (R55)	Non-preventable
Synovitis and tenosynovitis (M65)	Non-preventable
Systemic disorders of connective tissue in diseases classified elsewhere (M36)	Non-preventable
T23 - Burn and corrosion of wrist and hand	Non-preventable
T34 - Frostbite with tissue necrosis	Non-preventable
T43 - Poisoning by, adverse effect of and underdosing of psychotropic drugs, not elsewhere classified	Non-preventable
T44 - Poisoning by, adverse effect of and underdosing of drugs primarily affecting the autonomic nervous system	Non-preventable
T66-T78 - Other and unspecified effects of external causes	Non-preventable
T670 - Heatstroke and sunstroke	Preventable
T78 - Adverse effects, not elsewhere classified	Non-preventable
T80-T88 - Complications of surgical and medical care, not elsewhere classified	Non-preventable
T81 - Complications of procedures, not elsewhere classified	Non-preventable
T814 - Infection following a procedure	Non-preventable
T814XXA - Infection following a procedure, initial encounter	Non-preventable
T82 - Complications of cardiac and vascular prosthetic devices, implants and grafts	Non-preventable
T83 - Complications of genitourinary prosthetic devices, implants and grafts	Non-preventable
T84 - Complications of internal orthopedic prosthetic devices, implants and grafts	Non-preventable
T86838 - Other complications of bone graft	Non-preventable
Thoracic, thoracolumbar, and lumbosacral intervertebral disc disorders (M51)	Non-preventable
Thyroiditis (E06)	Non-preventable
Toxic effect of contact with venomous animals and plants (T63)	Non-preventable
Transplanted organ and tissue status (Z94)	Non-preventable
Traumatic amputation of elbow and forearm (S58)	Non-preventable
Tuberculosis of other organs (A18)	Non-preventable
Type 2 diabetes mellitus (E11)	Preventable
Typhoid and paratyphoid fevers (A01)	Non-preventable
U04 - Severe acute respiratory syndrome [SARS]	Non-preventable
U07 - Emergency use of U07	Non-preventable
U07 - Infections due to SARS-CoV-2	Non-preventable
Ulcerative colitis (K51)	Non-preventable
Umbilical hernia (K42)	Non-preventable
Unspecified abdominal hernia (K46)	Non-preventable
Unspecified acute lower respiratory infection (J22)	Non-preventable
Unspecified appendicitis (K37)	Non-preventable
Unspecified arthropod-borne viral fever (A94)	Non-preventable
Unspecified jaundice (R17)	Non-preventable
Unspecified malaria (B54)	Non-preventable
Unspecified maternal hypertension (O16)	Preventable
Unspecified renal colic (N23)	Non-preventable
Unspecified viral hemorrhagic fever (A99)	Non-preventable
Unspecified viral hepatitis (B19)	Non-preventable
Urethral stricture (N35)	Non-preventable
Urethritis and urethral syndrome (N34)	Non-preventable
V00-Y99 - External causes of morbidity	Non-preventable
V01 - Pedestrian injured in collision with pedal cycle	Non-preventable
V80-V89 - Other land transport accidents	Non-preventable
V86 - Occupant of special all-terrain or other off-road motor vehicle, injured in transport accident	Non-preventable
V89 - Motor- or nonmotor-vehicle accident, type of vehicle unspecified	Non-preventable
Varicose veins of lower extremities (I83)	Preventable
Vascular syndromes of brain in cerebrovascular diseases (G46)	Preventable
Ventral hernia (K43)	Non-preventable
Viral agents as the cause of diseases classified elsewhere (B97)	Non-preventable
Viral and other specified intestinal infections (A08)	Non-preventable
Viral infection of unspecified site (B34)	Non-preventable
Viral pneumonia, not elsewhere classified (J12)	Non-preventable
Vitamin B12 deficiency anemia (D51)	Preventable
W00-W19 - Slipping, tripping, stumbling and falls	Non-preventable
W01 - Fall on same level from slipping, tripping and stumbling	Non-preventable
w25 - Contact with sharp glass, initial encounter	Non-preventable
W50-W64 - Exposure to animate mechanical forces	Non-preventable
Whooping cough (A37)	Non-preventable
Y04 - Assault by bodily force	Non-preventable
Z03.8 - Medical observation and evaluation for suspected diseases and conditions	Non-preventable
Z20 - Contact with and exposure to communicable diseases	Non-preventable
Z30 - Encounter for contraceptive management	Preventable
Z40-Z53 - Encounters for other specific health care	Non-preventable
Z41 - Encounter for procedures for purposes other than remedying health state	Non-preventable
Z45 - Encounter for adjustment and management of implanted device	Non-preventable
Z4582 - Encounter for adjustment or removal of myringotomy device (stent) (tube)	Non-preventable
Z46 - Encounter for fitting and adjustment of other devices	Non-preventable
Z47 - Orthopedic aftercare	Non-preventable
Z48 - Encounter for other postprocedural aftercare	Non-preventable
Z51 - Encounter for other aftercare and medical care	Non-preventable
Z77-Z99 - Persons with potential health hazards related to family and personal history and certain conditions influencing health status	Preventable
Z80 - Family history of primary malignant neoplasm	Non-preventable
Z83 - Family history of other specific disorders	Non-preventable
Z85 - Personal history of malignant neoplasm	Non-preventable
Z8673 - Personal history of transient ischemic attack (TIA), and cerebral infarction without residual deficits	Preventable
Z87 - Personal history of other diseases and conditions	Non-preventable
Z90 - Acquired absence of organs, not elsewhere classified	Non-preventable
Z92 - Personal history of medical treatment	Non-preventable
Z94 - Transplanted organ and tissue status	Non-preventable
Z95 - Presence of cardiac and vascular implants and grafts	Preventable
Z96 - Presence of other functional implants	Non-preventable
Z960 - Presence of urogenital implants	Non-preventable
Z96621 - Presence of right artificial elbow joint	Non-preventable
Z9664 - Presence of artificial hip joint	Non-preventable
Z98 - Other postprocedural states	Preventable
Z99 - Dependence on enabling machines and devices, not elsewhere classified	Non-preventable
Zygomycosis (B46)	Non-preventable
